# Risk factors for carotid plaque formation in type 2 diabetes mellitus

**DOI:** 10.1186/s12967-023-04836-7

**Published:** 2024-01-04

**Authors:** Jin Chen, Wenwen Li, Jingzhu Cao, Yuhan Lu, Chaoqun Wang, Jin Lu

**Affiliations:** 1 Department of Endocrinology and Metabolism, Changhai Hospital, Naval Medical University, Shanghai, 200433 China; 2 Department of health, The affiliated hospital of Qingdao University, Qingdao, China

**Keywords:** Type 2 diabetes mellitus, Carotid plaque, Risk factors

## Abstract

**Object:**

Patients with type 2 diabetes mellitus (T2DM) are at higher risk of developing atherosclerosis. Previous studies have analyzed the factors associated with diabetic macrovascular disease, although whether these factors are applicable to T2DM patients with carotid atherosclerosis remains unclear. Therefore, the aim of this study was to investigate the risk factors for the formation of carotid atherosclerotic plaque in hospitalized T2DM patients and to provide a theoretical basis for early prevention and treatment of carotid atherosclerosis in these patients.

**Methods:**

A total of 949 patients with T2DM were included in the study. Carotid ultrasound identified 531 patients with carotid atherosclerotic plaque. The waist-to-hip ratio (WHR), blood glucose, liver and kidney function, blood lipid profile, islet function, and other indicators were measured at the same time to identify the risk factors and predictive significance of T2DM carotid plaque.

**Results:**

The proportions of men, diabetes nephropathy (DN) and hypertension in T2DM patients with carotid plaque are higher than those without carotid plaque(*P* < 0.05). Age, duration of diabetes, WHR, Postprandial glucose (PPG), lipoprotein (a) [Lip (a)], carcinoembryonic antigen(CEA) and estimated glomerular filtration rate (eGFR) in T2DM patients with carotid plaque were higher than those without plaque (*P* < 0.05). Age, WHR, duration of diabetes, hypertension, males, and Lip (a) were independent risk factors for T2DM patients with carotid plaque. Age, WHR, duration of diabetes, and Lip (a) had a higher AUC to predict T2DM with carotid artery plaque (AUC: 0.750, 0.640, 0.678, 0.552 respectively; *P all* < *0*.001). After constructing the logit (P) value of the above risk factors, the area under the ROC curve was 0.816 (0.789–0.842, *P* < 0.001).

**Conclusion:**

Age, WHR, duration of diabetes, hypertension, males, and Lip (a) levels are the main risk factors for the formation of carotid plaque in T2DM patients. Combining the above risk factors provides a better prediction of carotid plaque formation in T2DM.

## Introduction

Type 2 diabetes mellitus (T2DM) is a multi-etiological metabolic disease characterized by chronic hyperglycemia, which is caused by deficiencies in insulin secretion and/or utilization. With the continuous improvement of social and economic levels, the prevalence of T2DM has increased globally and shows a trend of developing in younger people [1]. The prevalence rate of diabetes is also increasing yearly. In 2018, the prevalence of T2DM was 12.4% in a national survey in China, with its complications having become an important burden on the health of Chinese people. Macrovascular disease is not only the main cause of disability in patients with T2DM, but also may lead to their death and therefore poses a serious threat to the life and health of these patients. Atherosclerosis (AS) is the pathological basis of complications in large vessels such as the coronary arteries, brain, kidney, and lower limb arteries, although it primarily involves the aorta. This may lead to coronary heart disease, cerebrovascular events, lower limb arteriosclerosis, and occlusive diseases. Cardiovascular disease (CVD) is the main cause of mortality in patients with T2DM, with these patients having a 2–4 times greater risk of CVD events than those without diabetes [2]. Chronic hyperglycemia is known to induce the formation of late glycation end products, resulting in vascular endothelial damage and decreased arterial wall elasticity. Chronic hyperglycemia can also lead to lipid metabolism disorders, oxidative stress, and inflammation, which form the pathological basis of AS. Carotid AS is a recognized marker of subclinical AS and a strong predictor of future clinical cardiovascular events [3]. Therefore, it is important to develop new methods for the early prediction of macrovascular complications in patients with T2DM. T2DM is associated with a higher risk of carotid plaque formation. The aims of this study were to analyze the main risk factors for carotid plaque occurrence in hospitalized T2DM patients with poor blood glucose control and to provide a theoretical basis for early prevention and treatment of diabetic great vessel lesions in these patients.

## Data and methods

### General information

A cross-sectional study was conducted on 949 patients with T2DM according to the WHO diagnostic criteria at Shanghai Changhai Hospital from January 2018 to January 2020. Subjects who met the following diagnostic criteria were included in the study: (1) Diagnostic criteria for T2DM: typical symptoms of polydipsia, polyuria, polyphagia, and weight loss, in addition to any of the following diagnostic criteria. ① fasting blood glucose ≥ 7.0 mmol/L; ②75 g glucose tolerance test 2 hours postprandial blood glucose ≥ 11.1 mmol/L; ③ random blood glucose ≥ 11.1 mmol/L. (2) the diagnostic criteria for T2DM patients with carotid plaque was identified by carotid ultrasound. Exclusion criteria: (1) acute complications such as lactic acidosis or diabetic ketoacidosis; (2) type 1 diabetes; (3) secondary diabetes mellitus; (4) severe heart, lung, and liver insufficiency; (5) mental diseases; (6) severe cerebrovascular diseases; (7) neoplasms or diseases of the blood system; (8) acute infection.

### Detection index and method

Basic clinical data, including sex, age, and diabetes course were collected after admission. On the second day after admission, patient’s height, weight, waist circumference, and hip circumference were measured after an overnight fast of 8 h, and then body mass index (BMI) and WHR were calculated. Two hours later, i.e. after 10 h fasting time, the patients underwent a blood test and a steamed bread meal test. Blood biochemistry and blood lipid profile were measured using an automatic biochemical instrument (Hitachi 7020), while insulin (F-Ins) and C-peptide (C-P) levels were determined by chemiluminescence (Roche). The homeostasis model of insulin resistance index was used to evaluate the insulin resistance(IR) status of the patients [(fasting blood glucose (mmol/L) × fasting insulin (mIU/L)]/22.5]. Carotid plaque was detected by an experienced doctor who was blinded to clinical follow-up data using B-mode ultrasound (Siemens Medical).

### Statistical analysis

The Kolmogorov-Smirnov test was used initially to test the normality of the distribution of measurement data. Normally distributed data represented by x ± s, while non-normally distributed data are expressed as the median (lower quartile, upper quartile). Independent samples t-test was used for comparison when data distribution was normal distribution and homogeneity of variance, otherwise, Mann-Whitney U test was analyzed. Categorical variables (gender, combined hypertension, and combined diabetic nephropathy) were described by frequency and rate, and were analyzed using the Chi-square test. Multivariate logistic regression was used to determine the odds ratio (OR) values and 95% confidence intervals of T2DM with carotid plaque. Receiver operating characteristic (ROC) curves were constructed and the area under the curve (AUC) was calculated to assess the predictive power of the independent risk factors. All statistical analyses were performed using the SPSS 20.0 statistical software package. *P* values < 0.05 were considered statistically significant.

## Results

### Clinical and biochemical characteristics

A total of 949 subjects with T2DM were enrolled in the study, including 531 T2DM subjects with carotid atherosclerotic plaque and 418 T2DM subjects without carotid atherosclerotic plaque. As shown in Table 1, patients with carotid plaque were older and had a longer duration of diabetes (*P* < 0.001). The proportion of males with carotid plaque was significantly higher than that without carotid plaque (*P* = 0.002). WHR, 2-hour postprandial blood glucose (PPG) levels, Lip (a), CEA and estimated eGFR in T2DM patients with carotid plaque were higher than those without carotid plaque (*P* < 0.001,except *P* = 0.006 for Lip (a) and P = 0.01 for PPG). While the proportion of hypertension and diabetes nephropathy (DN) in T2DM patients with carotid patients was significantly higher than that those without carotid plaque (*P* < 0.001) (Table 1).

**Table 1:** The clinical and laboratory characteristics of T2DM patients with (Plaque group) and without carotid plaque (Non-plaque group)

Variables	Non-plaque	Plaque	p value
n	418	531	
Male, n (%)	57.42%	67.23%	0.002
Hypertension, n (%)	167(167/418)	367(367/531)	<0.001
DN,n(%)	140(140/418)	229(229/531)	<0.001
Age, years	52.05 ± 12.15	62.32 ± 9.53	<0.001
BMI, kg/m^2^	25.49 ± 3.78	25.07 ± 3.75	0.088
WHR	0.92 ± 0.07	0.95 ± 0.06	< 0.001
Duration of diabetes, years	6(1,10)	11(5,19)	< 0.001
HOMA-IR	4.23 ± 1.76	4.88 ± 1.09	0.218
TC, mmol/L	4.70 ± 1.20	4.85 ± 1.30	0.074
TG, mmol/L	1.96 ± 0.79	1.83 ± 1.26	0.193
LDL-C, mmol/L	2.85 ± 1.03	2.95 ± 1.09	0.141
HDL-C, mmol/L	1.22 ± 0.61	1.20 ± 0.31	0.312
HDL-C/LDL-C	0.48 ± 0.26	0.46 ± 0.24	0.346
Residual cholesterol, mmol/L	0.53(0.34,0.84)	0.58(0.38,0.86)	0.129
Lip(a),mg/L	118.16 ± 42.73	145.81 ± 60.97	0.006
FBG,mmol/L	8.70 ± 3.31	8.67 ± 3.54	0.839
PPG,mmol/L	15.74 ± 3.90	16.60 ± 5.74	0.010
Uric acid, umol/L	327.45 ± 94.94	344.22 ± 94.85	0.277
eGFR, ml/min per 1.73m^2^	109.01 ± 53.20	95.14 ± 29.22	< 0.001
F-CP,ug/L	1.95 ± 0.91	1.99 ± 1.13	0.716
2 h-CP,ug/L	4.23 ± 2.11	4.30 ± 1.84	0.662
F-ins,mIU/L	7.10(4.10,11.95)	7.70(4.10,14.15)	0.126
2 h-ins,mIU/L	31.22 ± 10.46	31.67 ± 12.43	0.904
HbA1c,%	9.35 ± 2.79	9.34 ± 2.23	0.992
CEA,ng/ml	2.35(1.63,3.37)	2.85(1.95,4.23)	< 0.001
CA199,U/ml	8.51(4.31,17.05)	9.45(4.96,9.45)	0.120

*BMI* body mass index;* DN* diabetic nephropathy;* WHR* waist-to-hip ratio;* TC* total cholesterol;* TG* triglyceride;* LDL-C* Low density lipoprotein cholesterol;* HDL-C* High-density lipoprotein cholesterol; Lip(a):lipoprotein (a); HbA1c:glycosylated hemoglobin;* FBG* Fasting blood glucose;* PPG* Postprandial glucose;* CEA* carcinoembryonic antigen;* F-ins* Fasting insulin;* F-CP* Fasting C-peptide; 2 h-CP: C-peptide 2 h after meal;* 2 h-CP* C-peptide 2 h after meal

### Regression analysis of risk factors for carotid plaque in T2DM

**Fig. 1:**
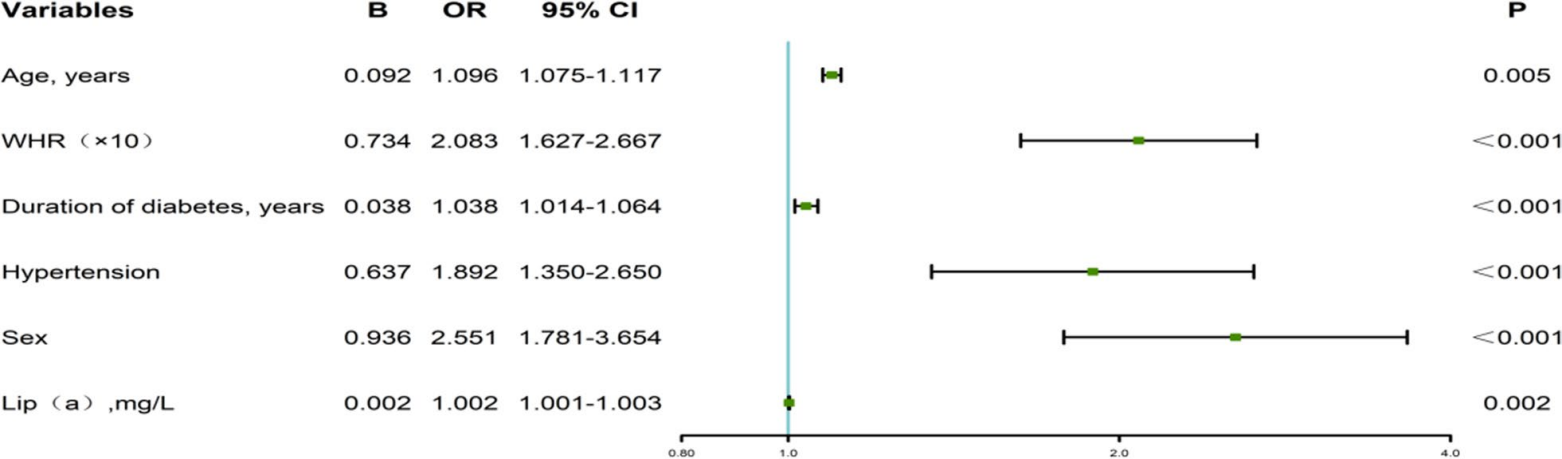
Forest map for predicting carotid plaque in T2DM patients

We further evaluated variables with different outcomes and analyzed their association with the risk of carotid plaque formation in T2DM using multivariate analysis. The results showed that age, WHR, duration of diabetes, hypertension, males, and Lip (a) were independent risk factors for T2DM patients with carotid plaque. All these variables correlated significantly with the formation of carotid plaque ( *P all* < 0.05) (Table 2; Fig. 1).

**Table 2:** Regression analysis of risk factors for carotid plaque in T2DM patients

Variables	B	OR value	95%CI	p value
Age, years	0.092	1.096	1.075–1.117	0.005
WHR(×10)	0.734	2.083	1.627–2.667	< 0.001
Duration of diabetes, years	0.038	1.038	1.014–1.064	< 0.001
Hypertension	0.637	1.892	1.350–2.650	< 0.001
Sex	0.936	2.551	1.781–3.654	< 0.001
Lip(a),mg/L	0.002	1.002	1.001–1.003	0.002

### ROC curve of carotid plaque formation in type 2 diabetes mellitus

**Fig. 2:**
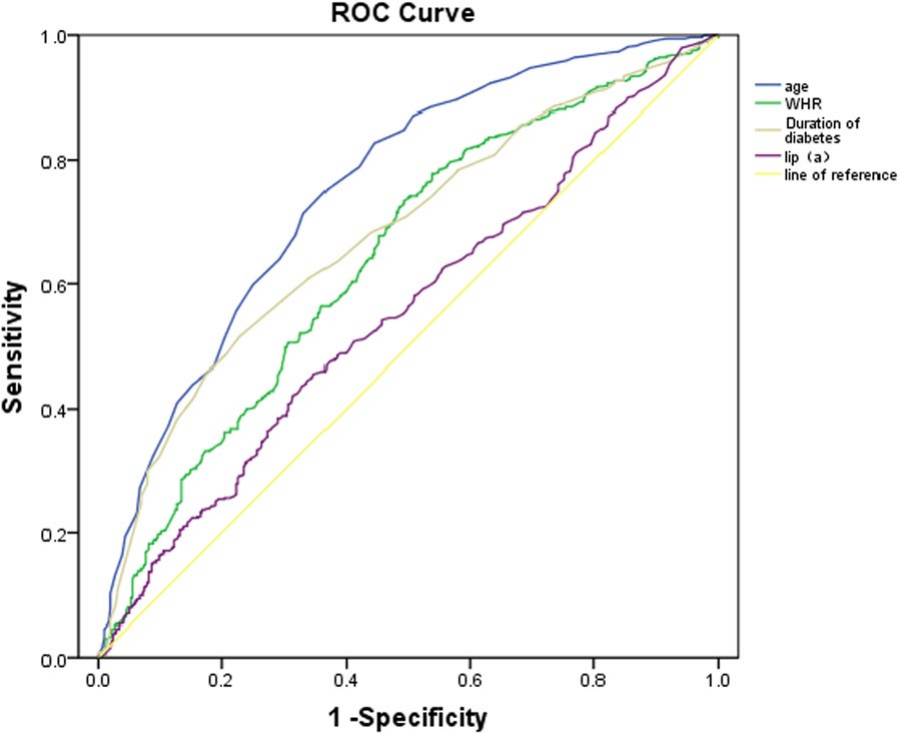
ROC curve of various risk factors for prediction of T2DM patients with carotid plaque

**Fig. 3:**
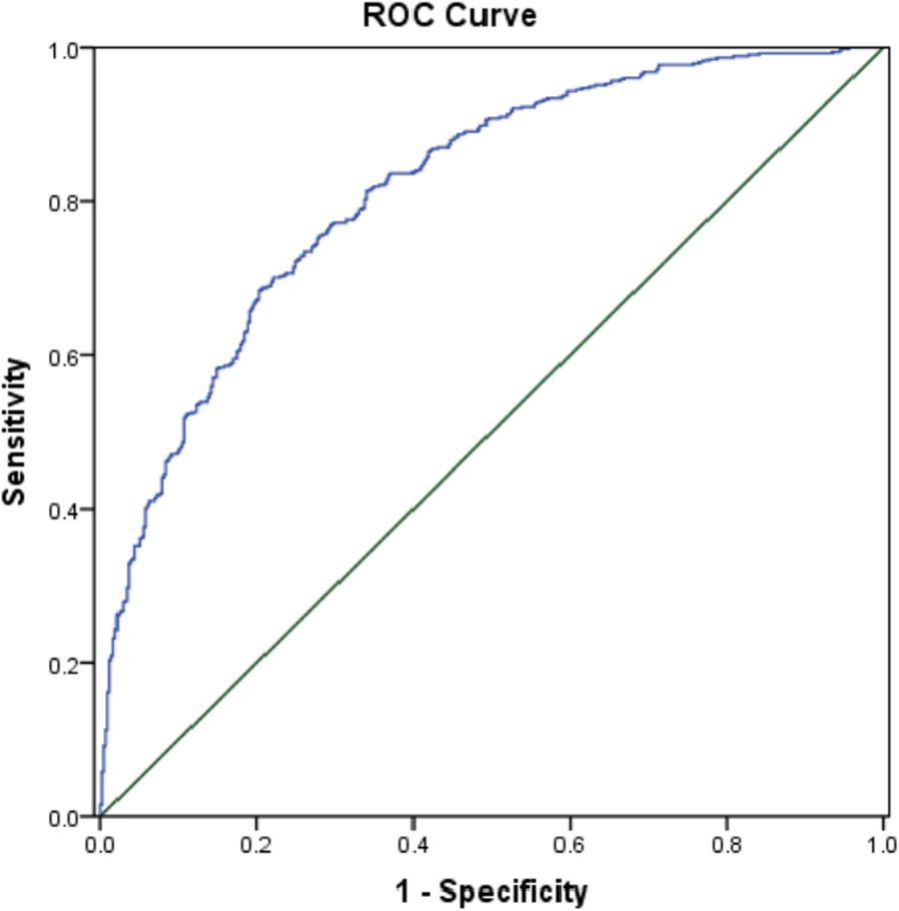
ROC curve of comprehensive risk factor for prediction of T2DM patients with carotid plaque

ROC curve analysis was performed using patient data to evaluate the predictive power of each risk factor. These analyses showed that age, WHR, duration of diabetes, and Lip (a) had a higher AUC to predict T2DM with carotid plaque (AUC: 0.750, 0.640, 0.678, 0.552 respectively; *P all* < 0.001). According to the results of logistic regression, we constructed logit (P) values and analysed the ROC curve, which showed that the prediction of T2DM with carotid plaque after combining the above risk factors had a high AUC (AUC: 0.816; *P* < 0.001). (Figs. 2 and 3).

## Discussion

Macrovascular complications are the main cause of death and disability in patients with T2DM, with their pathological basis being AS. Carotid AS is known to be the hallmark of systemic AS and is a strong predictor of cardiovascular events. It has been reported that the incidence of carotid plaque correlates significantly with the existence of AS, which closely reflects the overall severity of AS in the vascular system [4–7]. Endothelial injury and dysfunction caused by metabolic disorders associated with diabetes are important causes for initiating and promoting AS [8]. In this study, age, sex, WHR, hypertension, diabetic nephropathy, Lip (a), LDL-C, HDL-C, HDL-C/LDL-C, residual cholesterol, HbA1c, and the HOMA index were evaluated in hospitalized T2DM patients The relationship between IR, CEA, CA199, and carotid AS and the predictive power of each risk factor was evaluated. The results showed that inpatients with T2DM had poor blood glucose control with the mean HbA1c of the two groups being greater than 9%. T-test analysis showed that the blood glucose levels 2 h after a meal in patients with carotid artery plaque were significantly higher than that measured in patients without carotid artery plaque. The proportions of either male patients with carotid plaque or patients with hypertension were significantly higher in the group without carotid plaque. Previous studies have reported that low eGFR or high albuminuria are high-risk factors for cardiovascular events in patients with diabetes [9, 10]. Another observational study showed no significant association between eGFR level and atherosclerotic lesions in T2DM, although albuminuria was associated closely with AS in these patients [11].

The current study showed that eGFR in patients with carotid plaque was significantly lower than that in the group without carotid plaque, with the proportion of urinary microalbumin/creatinine ≥ 30 mg/g in the group with carotid plaque being significantly higher than that in the group without carotid plaque. However, eGFR and high albuminuria were not risk factors for carotid plaque after adjustment for confounding factors. Follow-up studies are therefore needed for further analysis of these relationships. We also found that the CEA level in the carotid plaque group was higher than that in the control group, although to date no correlation between these two variables has been reported. The specific mechanism, therefore, needs to be studied in greater detail. Multivariate analysis showed that age, duration of diabetes, blood pressure, WHR, and Lip (a) level were independent risk factors for T2DM patients with carotid artery plaque (*P* < 0.05). HDL is an anti-AS plasma lipoprotein and a protective factor of coronary heart disease, and is commonly known as a “vascular scavenger”. In this study, there was no significant difference in HDL-C levels in hospitalized T2DM patients. We observed that advanced age was a risk factor for carotid arteriosclerosis, again emphasizing age as a clinical risk factor for ASCVD events [12]. LDL-C level has always been the main lipid target for preventing CVD in patients with T2DM [13]. However, in the current study, there was no statistically significant difference between the two groups, although the mean value of LDL-C in patients without carotid plaque was close to 2.6 mmol/L while the value in patients with carotid plaque was greater than 1.8 mmol/L. From the perspective of the latest CDS guidelines, LDL-C combined with carotid plaque does not achieve the goal of control. The HDL-C/LDL-C ratio, a CVD predictor, reflects the protective and atherogenic lipoprotein balance [14] and is also considered a predictor of plaque vulnerability and coronary fatty plaque [15]. However, we observed no significant difference in the HDL-C/LDL-C ratio between the two groups. There is increasing evidence that Lip(a) may be a determinant of the risk of residual CVD when LDL-C is controlled at standard targets [16]. We, therefore, investigated the relationship between plasma Lip(a) levels and carotid AS in patients with T2DM. However, there have been conflicting results regarding the relationship between Lip(a) levels and CVD risk in patients with diabetes, with some studies reporting a positive correlation between carotid AS and Lip(a) levels in both the general population and patients with diabetes [17], whereas some studies have reported no significant correlation between the two variables [18]. For example, a large prospective study over 10 years showed only a moderately positive or no correlation [19] between Lip(a) levels and future CVD in patients with T2DM [20, 21]. The current study further confirmed the positive effect of Lip (a) in promoting carotid plaque formation, although the mechanism of this effect requires further investigation. Studies have suggested that oxidized Lip(a) is related to the promotion of an anti-fibrinolytic environment, foam cell formation, generation of fatty streaks, and an increase in smooth muscle cells, that together promote the formation of AS [22]. However, the specific mechanism of this effect needs to be further investigated in our follow-up studies. IR has been proved to be common before the onset of T2DM and plays an important role in the occurrence and development of AS [23, 24]. In the current study, no significant difference was observed in the HOMA index between the two groups, a result inconsistent with the conclusions of previous studies. This difference may be related to the non-onset patients and the influence of insulin and other drugs used by patients in our study. We also found that the WHR correlated strongly with carotid plaque load. There is evidence that an increased WHR is a key determinant of atherosclerotic burden in overweight subjects [25, 26] with studies also showing that the ratio is more strongly associated with all-cause mortality and incidence of myocardial infarction in obese subjects [27] than that of BMI. The pathophysiological association between abdominal obesity and CVD remains challenging. Adipocyte hypertrophy may be the first marker of mitochondrial dysfunction, which may lead to adipose tissue dysfunction and inflammatory response [28]. Previous studies have shown that WHR has good diagnostic consistency with abdominal fat measured by CT [29], although we did not use CT scans or whole-body dual energy X-ray absorptiometry to assess abdominal fat in our study. However, taken together, the above results show that patients with abdominal obesity and T2DM are at greater risk of developing AS. At the same time, our analysis found that the prediction of T2DM with carotid plaque after four risk factors of age, WHR, disease duration and Lip (a) was high (AUC: 0.816; P < 0.001).

Large vessel complications caused by AS are the main cause of disability and death in patients with T2DM. It is generally believed that the pathogenesis of diabetic vascular complications is caused by an imbalance between injury and endogenous protective factors. A variety of endogenous protective factors secreted by the endothelium, liver, skeletal muscle, and other tissues are believed to play an important role in countering diabetic damaging factors and maintaining vascular homeostasis. Some molecules, such as irisin and lipins, have recently been identified as new protective factors against diabetic AS, and the protective effect of HDL has also been reinterpreted. Our findings evaluated clinically measurable factors that predicted the risk of carotid plaque formation. Atherosclerosis is a multifactorial and complex process involving endothelial dysfunction, vascular inflammation, proliferation of vascular smooth muscle cells, thrombosis, monocyte expansion and differentiation into macrophages, and transformation of pathologically resident macrophages into foam cells [30].
